# Two-dimensional and three-dimensional T2 weighted imaging-based radiomic signatures for the preoperative discrimination of ovarian borderline tumors and malignant tumors

**DOI:** 10.1186/s13048-022-00943-z

**Published:** 2022-02-03

**Authors:** Xuefen Liu, Tianping Wang, Guofu Zhang, Keqin Hua, Hua Jiang, Shaofeng Duan, Jun Jin, He Zhang

**Affiliations:** 1 Department of Radiology, Obstetrics and Gynecology Hospital, Fudan University, Shanghai, P.R. China; 2 Department of Gynecology, Obstetrics and Gynecology Hospital, Fudan University, Shanghai, P.R. China; 3 GE Healthcare, Shanghai, P.R. China; 4 Department of Pathology, Obstetrics and Gynecology Hospital, Fudan University, Shanghai, P.R. China

**Keywords:** Ovarian neoplasm, Magnetic resonance imaging, Computer-Assisted Diagnosis, Radiomics

## Abstract

**Background:**

Ovarian cancer is the most women malignancy in the whole world. It is difficult to differentiate ovarian cancers from ovarian borderline tumors because of some similar imaging findings.Radiomics study may help clinicians to make a proper diagnosis before invasive surgery.

**Purpose:**

To evaluate the ability of T2-weighted imaging (T2WI)-based radiomics to discriminate ovarian borderline tumors (BOTs) from malignancies based on two-dimensional (2D) and three-dimensional (3D) lesion segmentation methods.

**Methods:**

A total of 95 patients with pathologically proven ovarian BOTs and 101 patients with malignancies were retrospectively included in this study. We evaluated the diagnostic performance of the signatures derived from T2WI-based radiomics in their ability to differentiate between BOTs and malignancies and compared the performance differences in the 2D and 3D segmentation models. The least absolute shrinkage and selection operator method (Lasso) was used for radiomics feature selection and machine learning processing.

**Results:**

The radiomics score between BOTs and malignancies in four types of selected T2WI-based radiomics models differed significantly at the statistical level (*p* < 0.0001). For the classification between BOTs and malignant masses, the 2D and 3D coronal T2WI-based radiomics models yielded accuracy values of 0.79 and 0.83 in the testing group, respectively; the 2D and 3D sagittal fat-suppressed (fs) T2WI-based radiomics models yielded an accuracy of 0.78 and 0.99, respectively.

**Conclusions:**

Our results suggest that T2WI-based radiomic features were highly correlated with ovarian tumor subtype classification. 3D-sagittal MRI radiomics features may help clinicians differentiate ovarian BOTs from malignancies with high ACC.

## Background

Ovarian borderline tumors (BOTs) account for approximately 10–15% of epithelial ovarian tumors, with an annual prevalence of 1.8–4.8/100,000 women worldwide [1]. Compared with other ovarian malignant tumors, ovarian BOTs often occur in young patients with early-stage disease, and patients have a good prognosis with fertility-sparing conservative treatments [2, 3] . Therefore, preoperative identification of patients with ovarian lesions suspected of being BOTs may be helpful in their management.

Magnetic resonance imaging (MRI) has many advantages in determining the etiology of ovarian masses and is widely used in clinical centers [4] . MRI has high diagnostic performance in differentiating between ovarian benign tumors and malignant tumors [5–9] . Considering the ability to discriminate BOTs from malignant epithelial ovarian tumors, conventional MRI varies with a sensitivity of 58% to 100% and a specificity of 61% to 100%, respectively [7, 10–13]. Functional MRI (for example, dynamic contrast-enhanced MRI, diffusion-weighted imaging and MR spectroscopy) showed a higher ability to distinguish a BOT from ovarian epithelial cancer than conventional MRI, such as T1-weighted imaging (T1WI) and T2-weighted imaging (T2WI), as shown in recently published studies [11, 12]. However, given that functional MRI acquisition is not routinely used in clinical scenarios, the scanning parameters are not presently standardized universally and may change across MRI machines or institutions. Gross morphological characteristic imaging features appreciated on T1WI and T2WI still have better applicability in the differentiation of BOTs from other malignancies.

As a research hotspot, radiomics is defined as a new ‘data-driven’ approach for extracting large sets of quantitative signatures from radiological images and shows its potential application in medicine [14, 15]. MR-based radiomic signatures has been shown to help to categorize tumor subtypes and assess tumor presence, spread, recurrence or response to treatment in female cancer patients [16–21]. To date, there have been limited MRI radiomics studies concerning ovarian BOT and epithelial cancer categorization. The purpose of this research was two-fold: first, we planned to evaluate the diagnostic performance of the MRI radiomics model in discriminating ovarian BOTs from malignancies; second, we sought to clarify whether three-dimensional MR-based radiomic signatures (of the whole lesion) could show better discriminative performance than two-dimensional radiomic signatures (of the maximum lesion) could in the same study sample.

## Patients and methods

### Patients

Our institutional review board (Gynecological and Obstetric Hospital, School of Medicine, Fudan University, Shanghai, China) approved this retrospective study, and the requirement for informed consent was waived for all participants. From January 2014 to December 2017, 438 consecutive patients with clinically suspected gynecological diseases were retrospectively retrieved from our institutional picture archiving and communication system (PACS, GE). The inclusion criteria were as follows: 1) patients with no previous pelvic surgery; 2) patients with no previous gynecological disease history; and 3) patients who had MRI examinations performed at our institution before pelvic or laparoscopic surgery. The exclusion criteria were as follows: 1) patients with previous pelvic surgical history or radiation history; 2) patients whose MRI data were unavailable either due to the examination being performed at another institution or due to claustrophobia; or 3) patients whose data lacked histological results. A total of 91 patients (average age, 39.8 ± 14.9 years) with pathologically proven ovarian borderline tumors and 105 patients with ovarian malignancies (average age, 51.9 ± 12.1 years) were selected as the study sample for signature selection (Table 1). The information on FIGO stage, pathological type, immunohistological staining results, and laboratory tests were collected through a hospital information system.

**Table 1:** The summary of the pathological types and numbers of the selected samples

Pathological type	Numbers	Age (yrs.)^a^
Ovarian borderline tumor	91	39.75 ± 14.85
Ovary malignancies	105	51.91 ± 12.05
Endometroid cancer	3	44.67 ± 6.02
Low-grade adenocarcinoma	3	42.33 ± 19.96
Clear cell type	5	49.4 ± 10.33
High-grade serous carcinoma	83	52.93 ± 11.28
Mucinous carcinoma	7	50 ± 16.33
Mixed carcinoma	4	50 ± 7.65
Total	196	46.26 ± 14.71

^a^mean ± standard deviation

### MR image acquisition and lesion segmentation and radiomics feature selection

**Fig. 1:**
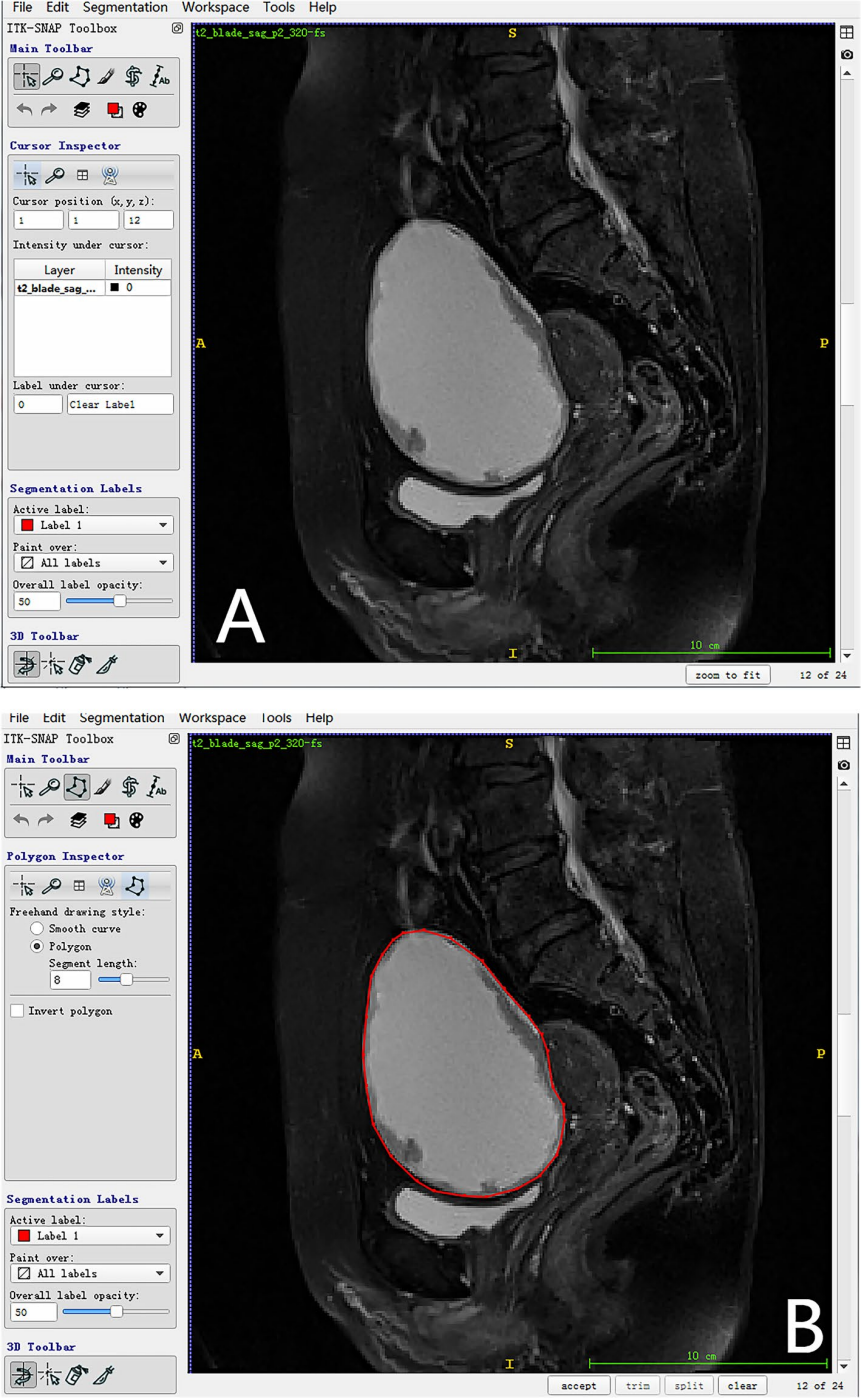
A 58-year-old woman with pathologically proved high-grade serous carcinoma. We selected the maximum lesion slice on sagittal fs-T2WI and segmented manually along the lesion margin with segmentation tool on ITK-SNAP software. The original fs-T2WI image (**A**) and region of interest selected image (**B**)

MRI was performed using a 1.5-T MR system (Magnetom Avanto, Siemens) with a phased-array coil. The routine MRI protocols used to assess pelvic masses included axial turbo spin-echo (TSE) T1-weighted imaging (T1WI), coronal TSE T2-weighted imaging (T2WI), and axial/sagittal TSE fat-suppressed T2WI (fs-T2WI). All lesion segmentation was performed by an experienced radiologist (H.Z.). The lesion segmentation on MRI was manually outlined using ITK-SNAP software (ITK-SNAP, version 3.4.0, www.itksnap.org) (Fig. 1). Two segmentation methods were used in this study: maximum lesion segmentation (two-dimensional, 2D) and whole-lesion segmentation (three-dimensional, 3D) on both sagittal fs-T2W images and coronal T2W images. In 2D segmentation, we chose one slice with the largest lesion diameter in two protocols as the premium picture for segmenting the whole lesion. In 3D segmentation, the entire lesion from both protocols was outlined and segmented slice by slice. After the tumor segmentation process, MR-based radiomics signatures were extracted from 2D/3D sagittal fs-T2W and 2D/3D coronal T2W images using AK software on a personal computer (Fig. 1).

### Image feature extraction and selection

A total of 396 radiomics features from the volume of interest were extracted automatically using in-house software (Analysis Kit, version 3.0.0, GE Healthcare). Thereafter, the whole dataset was randomly divided into two parts: a training cohort and a testing cohort. The radiomics score-based signatures were constructed with the least absolute shrinkage and selection operator (LASSO) method, which was used to select the most useful prognostic features in the training data set. A radiomics score (Rad-score) was computed for each patient through a linear combination of selected features weighted by their respective coefficients. These radiomics scores were first assessed in the training data set and then validated in the testing data set.

### Statistical analysis

First, two-sample t-tests were performed to compare MR-based signature values between ovarian BOT and ovarian cancer. Next, the sensitivity (SEN), specificity (SPE), positive predictive value (PPV), and negative predictive value (NPV) were calculated when the performance of the two methods was evaluated for their ability to identify ovarian malignancies. Additionally, receiver operating characteristic (ROC) curve analysis was performed to evaluate various MR-based signature diagnostic values in discriminating BOTs from malignancies. A value of *p* < 0.05 was considered statistically significant.

## Results

### Clinical characteristics in both the training and testing data sets

In this study, we included 91 ovarian borderline tumors and 105 ovarian malignancies (83 serous epithelial carcinomas, 7 mucinous carcinomas, 4 mixed carcinomas, 5 clear cell type carcinomas, 3 endometrioid carcinomas and 3 low-grade carcinomas, Table 1). There was no statistically significant difference found between the training and the validation data set in either clinical characteristics or pathological subtypes (Table 2).

**Table 2:** Clinical and pathological data summaries in both training and testing cohort

		Training group (*N* = 99)		Testing group (*N* = 97)		*P* value
Age (yrs.)		45.9 ± 13.35		46.64 ± 15.90		0.961
< *30*		17(17.2%)		14(14.4%)		
* 30–50*		35(35.4%)		40(41.2%)		
> *50*		47(47.5%)		43(44.3%)		
Ki-67 expression (%)		32.37 ± 28.01		25.05 ± 26.35		0.946
< *50*		59(67.0%)		74(84.1%)		
* 50–75*		20(22.7%)		5(5.7%)		
> *75*		9(10.2%)		9(10.2%)		
CA-125 level(IU/L)		553.32 ± 994.28		300.30 ± 452.27		0.000
< *35*		15(22.7%)		18(27.7%)		
* 35–200*		17(25.8%)		24(36.9%)		
* 200–500*		13(19.7%)		10(15.4%)		
> *500*		21(31.8%)		13(20.0%)		
Category						0.980
* Borderline tumor*		47(47.5%)		44(45.4%)		
* Malignancies*		52(52.5%)		53(54.6%)		
Endometroid cancer		2(2.0%)		1(1.0%)		
Low-grade adenocarcinoma		0(0.0%)		3(3.1%)		
Clear cell type		1(1.%)		4(4.1%)		
Serous carcinoma		45(45.5%)		38(39.2%)		
Mucinous carcinoma		2(2.0%)		5(5.2%)		
Mixed carcinoma		2(2.0%)		2(2.1%)		

### Identification results based on MRI-radiomics signatures

**Fig. 2:**
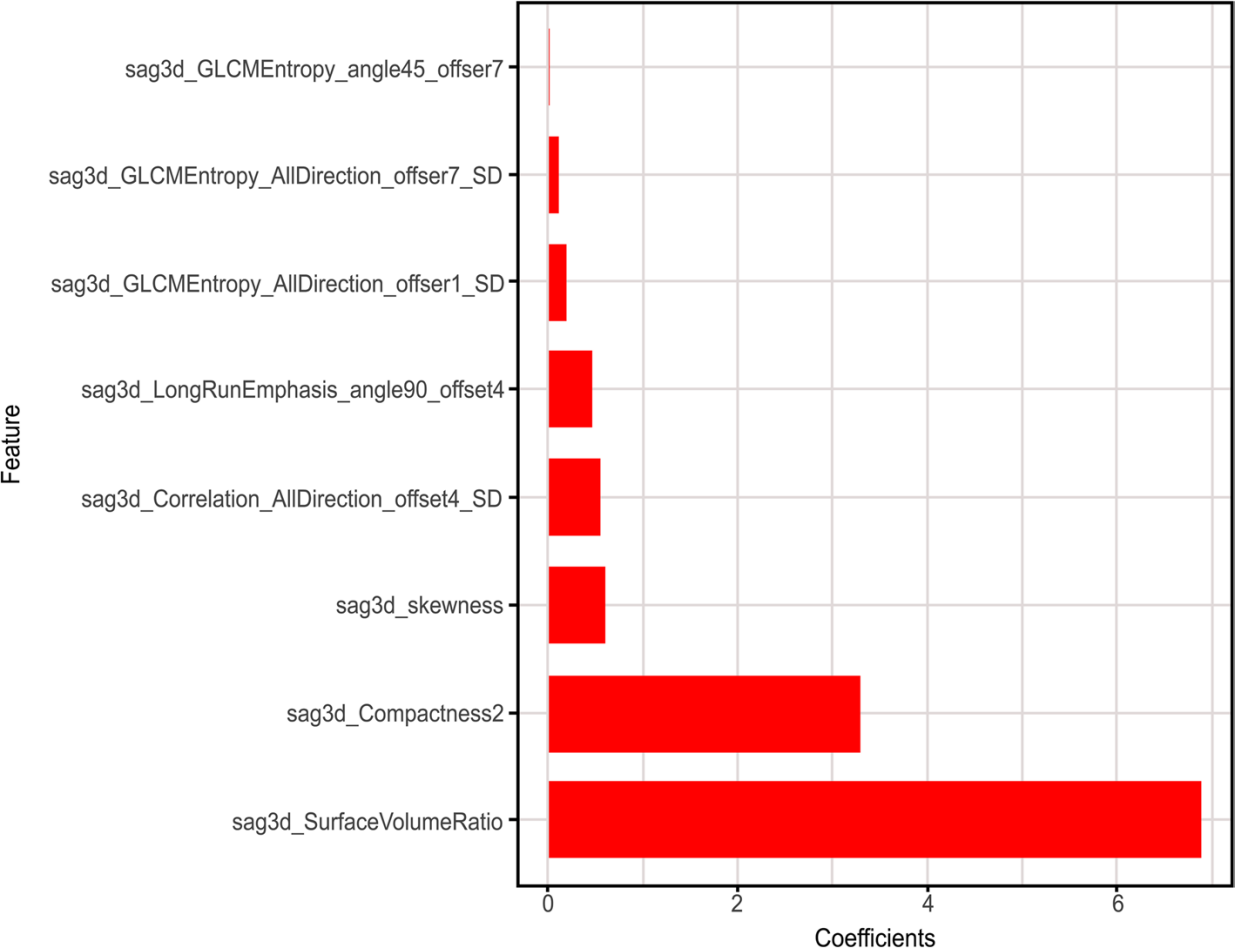
Histogram shows the weight of various features that contribute to the 3D signatures on sagittal fs-T2WI. The features that contribute to the radiomics signature model are displayed on the y-axis, with their coefficients in the LASSO analysis model dotted on the x-axis

**Fig. 3:**
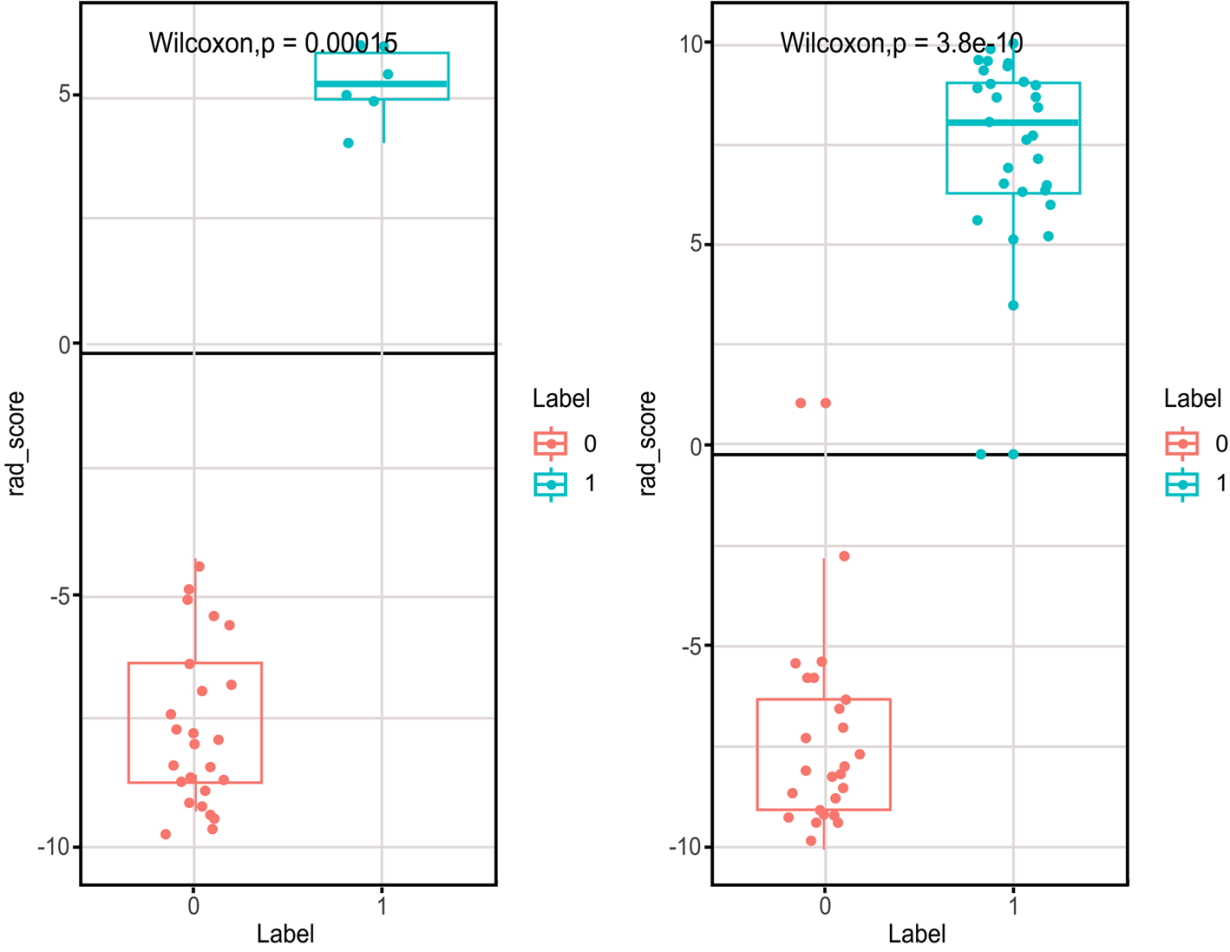
The Stem-and-leaf plots of the average Rad-score in the LASSO model using 3D fs-sagittal T2WI radiomics signatures. Training group (left) and Testing group (right)

The radiomics signature was weighted with the regression coefficients for the signature construction presented in the form of a histogram in Fig. 2. Overall, there was a statistically significant difference observed in the average Rad-score between BOTs (Fig. 3) and malignancies in each of the selected MR-based radiomics models (*p* < 0.0001, Table 3). Table 4 illustrates the final classification results of the training data set and the validation data set. The model was first determined on the training data set based on the area under the ROC curve (AUC). Then, we evaluated the model on the validation data set. The coronal MR-based radiomics segmentation model yielded an accuracy of 78.9% to 82.8%, while the sagittal model yielded an accuracy of 77.8% to 100%. The 3D sagittal MR-based radiomics model yielded an ACC and an AUC of as high as 100% in differentiating between BOTs and malignancies in the validation data set (Table 4).

**Table 3:** The average Rad-score between BOT and malignancies in various MR-based radiomics models

Model	BOT^a^	M	*P* value
2D Coronal Training	-0.73 ± 0.88	0.63 ± 0.58	< 0.0001
2D Sagittal Training	-0.63 ± 0.78	0.75 ± 0.93	< 0.0001
3D Coronal Training	-0.74 ± 0.66	0.87 ± 1.34	< 0.0001
3D Sagittal Training	-8.94 ± 2.15	9.55 ± 2.4	< 0.0001
2D Coronal Testing	-61.3 ± 295.1	0.22 ± 0.90	< 0.0001
2D Sagittal Testing	-0.19 ± 3.28	0.38 ± 2.99	< 0.0001
3D Coronal Testing	-0.66 ± 0.76	1.14 ± 1.41	< 0.0001
3D Sagittal Testing	-9.16 ± 2.65	8.89 ± 2.47	< 0.0001

^a^mean ± sd

**Table 4:** The diagnostic performance in differentiating malignancies from BOT based on various MR-based radiomics models

Model	Group	SEN	SPE	PPV	NPV	ACC	AUC(95% CI)
2d_cor	Training	0.708	0.936	0.919	0.759	0.821	0.90(0.85–0.96)
2d_cor	Testing	0.729	0.851	0.833	0.755	0.789	0.82(0.73–0.90)
3d_cor	Training	0.875	0.717	0.764	0.846	0.798	0.85(0.77–0.93)
3d_cor	Testing	0.936	0.717	0.772	0.917	0.828	0.84(0.76–0.93)
2d_sag	Training	0.776	0.902	0.884	0.807	0.840	0.89(0.83–0.96)
2d_sag	Testing	0.729	0.824	0.795	0.764	0.778	0.79(0.69–0.88)
3d_sag	Training	1.000	1.000	1.000	1.000	1.000	1.0(1.0–1.0)
3d_sag	Testing	1.000	0.980	0.980	1.000	0.990	1.0(1.0–1.0)

*SEN* sensitivity, *SPE* specificity, *PPV* positive predictive value, *NPV* negative positive value, *ACC* accuracy, *AUC* area under the curve, *CI* confidence interval

### Comparison of the performance results between the 2D and 3D radiomics models

**Fig. 4:**
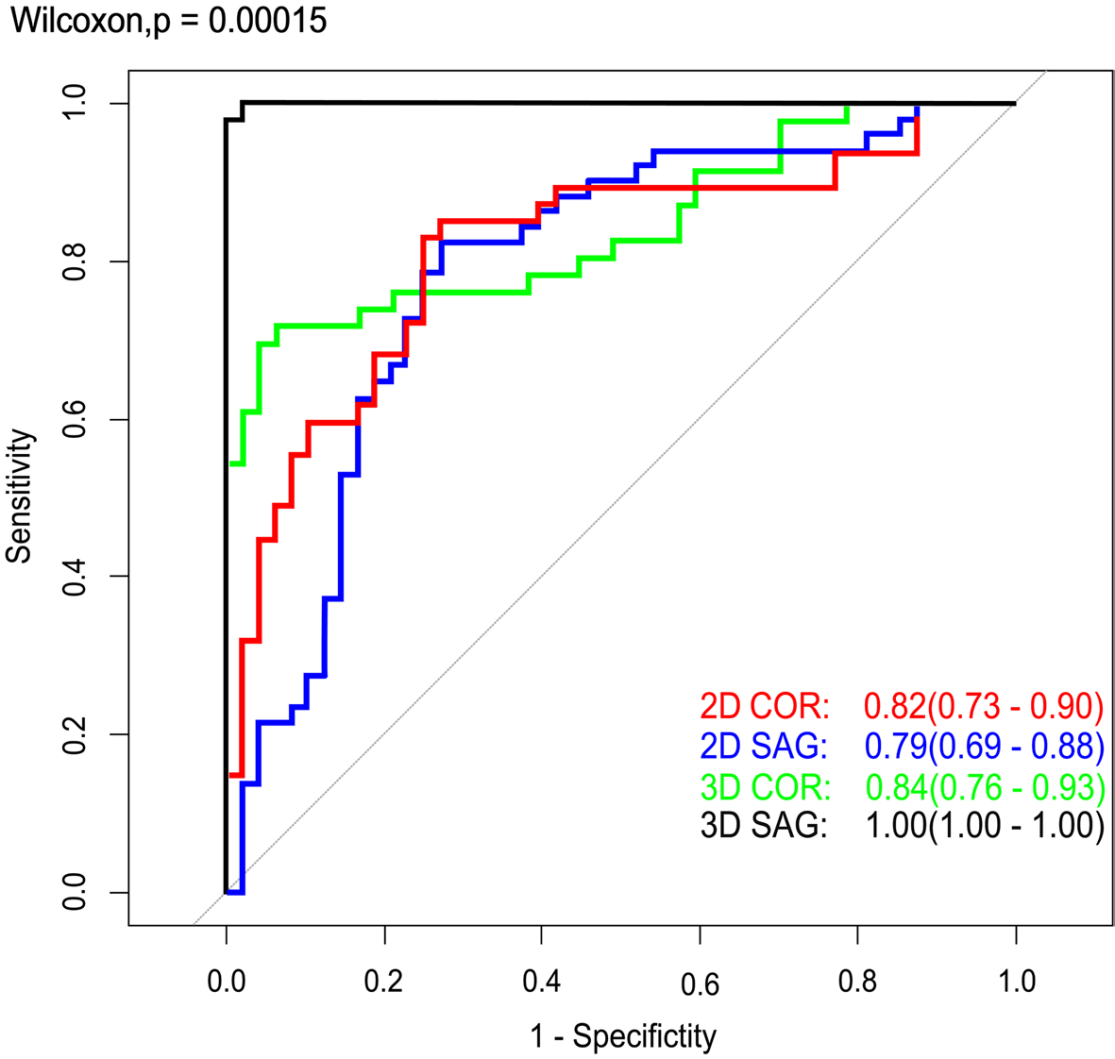
ROC analysis of four kinds of MR-based radiomics signature models in determining ovarian malignancies from BOTs. Training group (**A**) and Testing group (**B**)

Considering two acquisition protocols, both coronal and sagittal MR-based features showed competitive accuracy in discriminating BOTs from malignancies either in 2D or 3D segmentation mode (2D AUC: 0.82 versus 0.84 and 3D AUC: 0.79 versus 1.0, respectively). 3D sagittal fs-T2W images have the best performance compared to the other three methods in discriminating malignancies from BOTs, with an accuracy of 99% in the testing model. The ROC curve analysis with four kinds of segmentation methods in the validation group is summarized in Fig. 4.

## Discussion

Ovarian BOT is a type of low-potential epithelial tumor with a relatively good prognosis after treatment. Sometimes, it is difficult to discriminate BOTs from ovarian malignancies solely on imaging information due to some overlapping imaging findings between the two [22]. Our current results showed that the 3D MR-based radiomics signatures derived from sagittal fs-T2WI yielded an ACC of 100% in differentiating ovarian malignancies from BOTs and may help clinicians make a correct diagnosis before surgery. To the best of our knowledge, this is the first reported study focusing on the diagnostic performance of MR-based radiomics signatures in ovarian tumor classification with 2D and 3D segmentation methods.

In the present study, the 3D signatures showed better performance than the 2D signatures did. This result can be easily appreciated because the 3D model utilized information of the whole lesion, more truly reflecting the tumoral heterogeneity than the 2D model did. The current result is contrary to the previous CT radiomics study in which 2D radiomics features performed slightly better in non-small cell lung cancer prognostic estimation than 3D did [23]. The authors concluded that the reason might be related to the various axial CT image resolutions in their study in which the training and validation cohorts in the study sample were selected from different institutions.

Considering the two selected MRI protocols, the fs-sagittal sequences performed better than coronal sequences did on both 2D and 3D segmentation methods. Of note, the 3D-sagittal MR radiomics model yielded ACCs of 100% and 99% in the training and testing groups, respectively. This finding is in accordance with our previous study in which fs-T2WI was also superior to coronal T2WI in Type I and Type II ovarian cancer categorization [5]. We believe that the sharp contrast between the lesion and the background on the fs MR sequence may play a role in the final determination. However, the true mechanism is unclear, and this result should also be validated in a future study with a large study sample.

Several radiomics studies using CT images have been reported for ovarian mass classification and prognostic estimation [24–27]. Fathi et al. found that the time-to-peak and wash-in rate parameters showed a high SEN (89% for the linear discriminant analysis [LDA] classifier and 97% for the support vector machine [SVM] classifier) and a high SPE (93% for LDA and 100% for SVM) in distinguishing malignancies from benign ovarian conditions among 55 sonographically indeterminate ovarian masses [26]. Qiu et al. acquired two sets of CT images (pretreatment and posttreatment) to compare three image features (tumor volume, tumor density, and density variance) between the two image sets in 30 ovarian cancer patients, and their model achieved an area under the curve of 0.831 in predicting progression-free survival when combining all three features together [25]. In this study, we used the LASSO method to establish the radiomics features model during the radiomics signature selection step as well as during the machine learning process. The Lasso model is reportedly a suitable method for analyzing a small sample with high-dimensional features due to its advantage of avoiding overfitting. A similar method was also reported in two recently published studies with promising results [18, 28].

There remains a limited number of studies on MR-based radiomics in ovarian tumor classification and posttreatment response prediction. In one study with 22 patients with advanced ovarian cancer, the authors found that apparent diffusion coefficient (ADC) values derived on the ADC map between primary ovarian cancer and metastatic sites differed significantly and may be used as response markers [29]. In the present study, we did not include DW images in the texture analysis. The lesion resolution on DWI, especially with large lesions, is relatively low, which is sometimes difficult to precisely outline in postprocess software. Moreover, in our previous study, we did not find that the ADC map could contribute more useful signatures in task classification than conventional MR images (T1W and T2W images) could [5]. Compared with traditional MRI analysis in differentiating BOTs from malignancies, radiomics signature results show better performance. In a traditional MRI reading session, the imaging signs always overlap with each other to some extent (for example, large size, solid components, irregular and thick septa) and lead to an inaccurate diagnosis [27, 30–32]. A recent study with proton MR spectroscopy (MRS) reported that the SEN and SPE were 91% and 100% for solid components, respectively; additionally, the SEN and SPE were 84% and 82% for cystic components, respectively [12]. However, MRS scans are highly unit-dependent and time-consuming examinations and require operators with more experience than conventional methods do. From this point of view, radiomics signature analysis shows the potential clinical application owing to its simple segmentation step.

The limitations of this study included the fact that we did not include contrast-enhanced (CE) MR images to establish the MRI radiomics model. The CE-MRI scan was not available for all included patients in the current study, and therefore, we did not select this protocol for analysis to diminish the selection bias. Furthermore, in the present study, we only used conventional T2WI to establish a radiomics diagnostic model, which is different from the clinical reading scenario (mostly including T1WI, T2WI and DWI). Further study is necessary to explore the difference between one acquisition sequence and multiple acquisition sequences as in the clinical setting. In addition, all segmentation procedures were manually outlined on T2WI showing the best of the lesion; however, it is still an operator-dependent procedure, and interoperator variation in segmentations may be emphasized, especially with multiple sequence images. Finally, all MR images were acquired in a 1.5-T MRI scanner, and a comparison study between 1.5-T and 3.0-T MRI machines should be validated in a large study in the future.

In summary, our results suggest that radiomics features that were extracted from T2W images were highly correlated with ovarian tumor subtype classification. 3D fs-sagittal MRI radiomics features may help clinicians differentiate ovarian BOTs from malignancies with high ACC.

## Data Availability

The authors declare that all data supporting the findings of this study are available within the article.

## Abbreviations

BOT: Borderline tumor
2D: Two-dimensional
3D: Three-dimensional
DWI: Diffusion weighted imaging
ADC: Apparent diffusion coefficient
PACS: Picture archiving and communication system
LASSO: Least absolute shrinkage and selection operator
LDA: Linear discriminant analysis
SVM: Support vector machine
